# Fatty Acid Composition and Lipid Profile of *Diospyros mespiliformis, Albizia lebbeck*, and *Caesalpinia pulcherrima* Seed Oils from Nigeria

**DOI:** 10.1155/2014/283614

**Published:** 2014-10-16

**Authors:** Adewale Adewuyi, Rotimi Ayodele Oderinde

**Affiliations:** ^1^Department of Chemical Sciences, Redeemer's University, Mowe, Nigeria; ^2^Centre for Lipid Research, India Institute of Chemical Technology, Hyderabad 500 007, India; ^3^Industrial Unit, Department of Chemistry, University of Ibadan, Ibadan, Nigeria

## Abstract

The screening of lesser-known underutilized seeds as source of food has been a way of finding solution to food insecurity in developing nations. In this regard, oil as a class of food was extracted from the seeds of *Diospyros mespiliformis*  (4.72 ± 0.2%), *Albizia lebbeck*  (6.40 ± 0.60%), and *Caesalpinia pulcherrima*  (7.2 ± 0.30%). The oils were finally analyzed for their fatty acid composition, lipid classes, fatty acid distribution in the lipid fractions, and molecular speciation of the triacylglycerols, glycolipids, and phospholipids. The fatty acid composition of the oils varied with C18:2 fatty acid being the most dominant in the oils. Neutral lipids were the most abundant lipid class found in the oils while molecular species of the triacylglycerol with equivalent carbon chain number C40 was majorly present in the oils of *Diospyros mespiliformis* and *Caesalpinia pulcherrima*. The present study presents lesser-known underutilized seeds as possible sources of food.

## 1. Introduction

In time past, food insecurity has been a fundamental problem confronting most developing countries of the world. Access to adequate food has been a challenge because of the high level of poverty in developing nations of the world. As a result of this, there are concerted efforts to improve food production in some of these countries, but the populace still suffer from malnutrition due to certain constringent factors [1, 2]. Apart from inadequate food storage, production, and processing, the demand on food as industrial feed stock by local upcoming industries has also posed serious problem of food insecurity in such regions of the world. Oil and fat play vital role as a source of nutrient for both human and animals. Besides being food, they are also a functional means of industrial feed stock for the production of other industrial and domestic products. In South-East Asia, Europe, United States, and China, palm oil, rapeseed oil, and transgenic soybeans are well known conventional seed oils used to produce biodiesel. These oils are edible and can serve as food. Their use as feed stock for the production of biodiesel poses a threat on food security as this has also led to increase in their price in the market making them unaffordable for poor and low income earners. These well known conventional seed oils have also found applications in cosmetic, polymer, pharmaceuticals, and oleochemical industries.

Over the years, a number of researchers have investigated the potential suitability of plant seed species in serving as replacement for some of these well known conventional seed oils. A few of these oil bearing plant seeds have fruits which have been documented for their nutritional importance [3]. Recently, interest has been on the search for lesser known underutilized oil bearing seeds that can serve as source of oil to replace or serve as alternative for the well known conventional seed oils in market which are expensive to afford.

To address this problem of food insecurity there is need to screen the lesser-known plant seeds for their possible use. In this regard, attention has been focused on underutilised local seeds for possible development and use. There are several underexploited plant seeds in Nigeria with little information about their composition; examples of such plant seeds include* Diospyros mespiliformis*,* Albizia lebbeck,* and* Caesalpinia pulcherrima*. The plants are found throughout the West African Region. They are usually planted as a shade tree.* Diospyros mespiliformis* is a tree which is 30 m high with a straight bole to over 2 m in girth; the antibacterial and antifungal activities of the compounds isolated from the root have been reported [4, 5]. The fruit of* Diospyros mespiliformis* has been found edible as this is used in fermented drink and other meals [6]. The physicochemical properties of* Caesalpinia pulcherrima* and* Albizia lebbeck* seed oils have been previously reported [7]. They were reported to be good sources of carbohydrates, proteins, fat-soluble vitamins, and minerals [7] but the fatty acid composition, fatty acid distribution in the lipid classes, and the molecular speciation of the triacylglycerol as well as the glycolipid and phospholipids contents of the seed oils of these plants have not been well studied and reported. In continuation of our search for viable seed oils with economic importance, the present study is aimed at studying the composition and possible uses of some selected Nigerian seed oils (*Albizia lebbeck*,* Caesalpinia pulcherrima*, and* Diospyros mespiliformis*).

## 2. Materials and Methods

### 2.1. Materials

The seeds (*Diospyros mespiliformis*,* Albizia lebbeck*, and* Caesalpinia pulcherrima*) were harvested from the botanical garden of University of Ibadan, Oyo State, Nigeria. They were identified at the Herbarium Unit, Botany Department of the University of Ibadan. The seeds were manually separated from their pods, ground separately in a laboratory mill, and stored in a cellophane bag at 4°C prior to analysis. Solvents and chemicals used in this study were of analytical grade and were purchased from S.D. Fine Chemicals, Mumbai, India. Silica coated Tin Layer Chromatography (TLC) plates (20 × 20 cm) were procured from Sigma-Aldrich, Chemical Co., Steinheim, Germany.

### 2.2. Extraction and Fatty Acid Composition of the Oils

Oil was extracted from the seeds of* Diospyros mespiliformis*,* Albizia lebbeck*, and* Caesalpinia pulcherrima* as described by Adewuyi et al. [8]. The oils obtained were analyzed for their fatty acid composition as previously described [9]. Briefly, the fatty acids were determined as fatty methyl esters of the oils which were prepared by refluxing the oils at 70°C for 3 h in 2% sulphuric acid in methanol. Identification of the fatty acids and their composition was carried out on Agilent 6890 N series gas chromatography equipped with FID detector on a split injector. A fused silica capillary column (DB-225, 30 × 0.32 m i.d., J & W Scientifics, USA) was used with the injector and detector temperature maintained at 230°C and 250°C, respectively. The oven temperature was programmed at 160°C for 2 min and finally increased to 230°C at 4°C/min. The carrier gas was nitrogen at a flow rate of 1.5 mL/min. The area percentages were recorded with a standard Chemstation Data System.

### 2.3. Separation of Lipid Classes and Fatty Acid Distribution

The oils were separated into the different lipid classes (neutral lipids, glycolipids, and phospholipids) on a 1 g scale using silica gel open column chromatography as described by Christie [10]. Neutral lipids, glycolipids, and phospholipids were eluted successively using chloroform, acetone, and methanol, respectively. The lipid fractions were screened by TLC for the identification of components using hexane: ethyl acetate (90 : 10, v/v) as developing solvent for neutral lipids, chloroform: methanol: water (65 : 25 : 4, v/v/v) for glycolipids and phospholipids. The eluted spots were identified using different spray reagents such as iodine vapours for neutral lipids, ammonium molybdate and perchloric acid for phospholipids, and *α*-naphthol for glycolipids [11]. The individual fractions were pooled, distilled under vacuum to remove solvent, and weighed for quantification. The individual lipid fractions were converted into fatty acid methyl esters by refluxing with 2% sulphuric acid in methanol for 3 h. The methyl esters were later analyzed for fatty acid composition using GC.

### 2.4. Identification of Unsaponifiable Matters in the Seed Oils

This was determined according the method of Adewuyi et al. [12]. Briefly, oil was refluxed for 1 h in 25 mL of 2 M ethanolic potassium hydroxide. The reaction mixture was later diluted to 150 mL with distilled water and transferred into a separating funnel and the unsaponifiable matters were then extracted three times with 50 mL diethyl ether. The ether extract was first washed with 100 mL aqueous solution of 0.5 M potassium hydroxide in order to remove any residual fatty acids. This was further washed with distilled water until it was free of potassium hydroxide, dried over anhydrous sodium sulphate, and concentrated using a rotary evaporator. The unsaponifiables were identified by GC-MS using Agilent (Palo Alto, USA) 6890N gas chromatography equipped with an HP-1 MS capillary column connected to an Agilent 5973 mass spectrometer operating in the EI mode (70 ev; *m*/*z* 50–550; source temperature 230°C and quadruple temperature 150°C). Structural assignments were made based on interpretation of mass spectrometric fragmentation and confirmation by comparison of retention time as well as fragmentation pattern of authentic compounds and the spectral data obtained from the Wiley and NIST libraries.

### 2.5. Molecular Speciation of the Triacylglycerols of the Oils

The molecular speciation of the oils was determined using a reversed phased HPLC analysis on HP-1100 series HPLC equipped with an Evaporative Light Scattering Detector (ELSD) 2000 (Alltech ELSD 2000, Alltech Associates Inc, USA). About 25 *μ*L of triacylglycerols (1 mg/mL) was injected in the SGERP-column (250 SS 4.6-W5C18-RS). The molecular species of the triacylglycerols were eluted within 10 min using an isocratic mobile phase of 95 : 5 (v/v) of acetone/isopropanol at a flow rate of 1 mL/min. The molecular species were identified by their Equivalent Carbon Numbers (ECN), by injecting reference triacylglycerols mixture and also by comparing with the literature data. The operating conditions for ELSD are drift tube, temperature 30°C, and flow of nitrogen 1.5 L/min with impactor “on” mode.

## 3. Results and Discussion

### 3.1. Extraction and Fatty Acid Composition of the Oils

The oil yield of* Diospyros mespiliformis* was found to be 4.72 ± 0.2% while that of* Albizia lebbeck* (6.40 ± 0.60%) and* Caesalpinia pulcherrima* (7.2 ± 0.30%) was as previously reported [7]. The fatty acid composition of the seed oils varied as shown in Table 1.

C12:0 and C14:0 fatty acids were found only in* Diospyros mespiliformis* (0.84 ± 0.10 g/100 g fatty acids and 0.82 ± 0.10 g/100 g fatty acids, resp.) and* Caesalpinia pulcherrima* (0.2 ± 0.05 g/100 g fatty acids and 0.2 ± 0.02 g/100 g fatty acids, resp.). C20:1 was only found in* Albizia lebbeck* (0.3 ± 0.05 g/100 g fatty acids) and* Caesalpinia pulcherrima* (0.10 ± 0.03 g/100 g fatty acids) just as C22:1 was only present in* Albizia lebbeck* (6.30 ± 0.10 g/100 g fatty acids). C18:2 was recorded to be the most abundant fatty acid in* Diospyros mespiliformis* (34.97 ± 0.40 g/100 g fatty acids),* Albizia lebbeck* (47.2 ± 0.50 g/100 g fatty acids), and* Caesalpinia pulcherrima* (49.8 ± 0.30 g/100 g fatty acids). Among the three oils analyzed* Albizia lebbeck* (75.3 g fatty acids) had the highest amounts of unsaturated fatty acids, followed by* Caesalpinia pulcherrima* (68.7 g fatty acids) and* Diospyros mespiliformis* (60.14 g fatty acids). The oils are not good sources of long chain fatty acids (C20:0, C20:1, C24:0, and C24:1) as these were found in small amounts. Previous work of Chivandi et al. [13] reported C16:0 and C18:2 fatty acids to be high in* Diospyros mespiliformis*.

### 3.2. Separation of Lipid Classes and Fatty Acid Distribution

The oils were separated into neutral lipids, glycolipids, and phospholipids as presented in Table 2. The most dominant lipid class in the oils was neutral lipids. The distribution of the different fatty acids along the lipid classes is presented in Table 3. The neutral lipid was 92.50 ± 0.40% in* Albizia lebbeck*, 95.30 ± 0.50% in* Caesalpinia pulcherrima,* and 93.60 ± 0.20% in* Diospyros mespiliformis*. The fatty acids are distributed along the triglyceride bonds in different proportions with the phospholipids having the highest amounts of saturated fatty acids except in* Caesalpinia pulcherrima*. It was also observed that the amounts of saturated fatty acid slightly increased in the glycolipids. In* Diospyros mespiliformis*, C16:1 was only detected in the neutral lipids (0.41 ± 0.05 g/100 g fatty acids) and glycolipids (0.36 ± 0.05 g/100 g fatty acids) but not in phospholipids. The lipid classes accumulated C18:2 fatty acid in high amounts making these lipid classes good sources of this fatty acid. The long chain fatty acids in* Albizia lebbeck* were found to be the highest in the neutral lipids except for C24:0 which was higher in the glycolipids (3.6 ± 0.20 g/100 g fatty acids) and phospholipids (3.5 ± 0.10 g/100 g fatty acids) than in the neutral lipids (1.6 ± 0.20 g/100 g fatty acids).

### 3.3. Identification of Unsaponifiable Matters in the Seed Oils

The unsaponifiable matters were determined using GC-MS. The result is presented in Table 4. Vitamin E and phytol were determined in both* Albizia lebbeck* and* Caesalpinia pulcherrima*. Beta-amyrin, gamma-sitosterol, and octadiene were found only in* Diospyros mespiliformis*. Stigmasterol was found in* Caesalpinia pulcherrima* just as stigmastadiene was detected in* Albizia lebbeck*. Other compounds found in the unsaponifiables included hydrocarbons.

### 3.4. Molecular Speciation of the Triacylglycerols of the Oils

Triglycerides are esters derived from glycerol and three fatty acids. They are the major constituents of vegetable oils [14]. The molecular species were reported based on classification of triglycerides containing most abundant fatty acids as shown in Table 5. The most abundant molecular species in* Albizia lebbeck* are LnLnLn (33.27 ± 0.5%) and LLnLn (33.86 ± 0.2%), while in* Caesalpinia pulcherrima*, it was LLLn/LnLnP/LnLnO (42.63 ± 0.3%) and in* Diospyros mespiliformis*, it was 41.11 ± 0.50%. Molecular species with equivalent carbon chain number C48 (SLnS/OOO/POP) was found the least in all the three oils studied; it was 2.00 ± 0.2% in* Albizia lebbeck*, 1.00 ± 0.10% in* Caesalpinia pulcherrima*, and 1.86 ± 0.05% in* Diospyros mespiliformis*. The oil of* Albizia lebbeck* is a good source of LnLnLn and LLnLn,* Caesalpinia pulcherrima* is a good source of LLnLn and LLLn/LnLnP/LnLnO, while* Diospyros mespiliformis* is a good source of LLnLn and LLLn/LnLnP/LnLnO.

## 4. Conclusion

Seed oils of* Diospyros mespiliformis*,* Albizia lebbeck*, and* Caesalpinia pulcherrima* are lesser-known and underutilized. These oils were analyzed for their fatty acid composition, lipid classes, fatty acid distribution in the lipid fractions, and molecular speciation of the triacylglycerols. The result showed C18:2 fatty acid as the dominant fatty acid in the oils of* Diospyros mespiliformis*,* Albizia lebbeck*, and* Caesalpinia pulcherrima*. Neutral lipids were the dominant lipid class in the studied oils while the amounts of the glycolipids species varied in the different oils. Molecular species with equivalent carbon chain number C40 was majorly present in the oils of* Diospyros mespiliformis* and* Caesalpinia pulcherrima*, while the different glycolipids and phospholipids analysed varied in the oils. The fatty acid composition and lipid profile of these seed oils present them as possible potential industrial resources.

## Figures and Tables

**Table 1:** Fatty acid composition (g/100 g fatty acids) of *A. lebbeck, C. pulcherrima*, and *D. mespiliformis* seed oils.

Fatty acids	*A. lebbeck *	*C. pulcherrima *	*D. mespiliformis *
12:0	ND	0.2 ± 0.05	0.84 ± 0.10
14:0	ND	0.2 ± 0.02	0.82 ± 0.10
16:0	15.0 ± 0.50	16.3 ± 0.30	22.46 ± 0.30
16:1	0.9 ± 0.10	1.3 ± 0.10	0.42 ± 0.10
18:0	4.1 ± 0.10	11.6 ± 0.20	11.35 ± 0.20
18:1	18.3 ± 0.40	17.0 ± 0.50	24.23 ± 0.30
18:2	47.2 ± 0.50	49.8 ± 0.30	34.97 ± 0.40
18:3	0.4 ± 0.10	0.4± 0.30	0.52 ± 0.10
20:0	3.3 ± 0.10	1.4 ± 0.10	1.46 ± 0.10
20:1	0.3 ± 0.05	0.10 ± 0.03	ND
22:0	0.2 ± 0.00	0.8 ± 0.10	1.67 ± 0.30
22:1	6.30 ± 0.10	ND	ND
24:0	2.1 ± 0.10	0.8 ± 0.05	1.26 ± 0.20
24:1	1.9 ± 0.05	0.1 ± 0.00	ND
Unsaturated	75.3	68.7	60.14

Saturated	24.7	31.3	39.86

Values are mean ± standard deviation of triplicate determinations. ND = not detected.

**Table 2:** Lipid profile of *A. lebbeck, C. pulcherrima,* and *D. mespiliformis* seed oils.

Sample	NL	GL	PL
*A. lebbeck *	92.50 ± 0.40	6.80 ± 0.10	0.70 ± 0.10
*C. pulcherrima *	95.30 ± 0.50	4.60 ± 0.10	0.10 ± 0.00
*D. mespiliformis *	93.60 ± 0.20	6.10 ± 0.30	0.30 ± 0.05

NL = neutral lipids, GL = glycolipids, and PL = phospholipids.

**Table 3:** Fatty acid compositions (g/100 g fatty acids) in the lipid classes of *A. lebbeck*, *C. pulcherrima*, and *D. mespiliformis*.

Fatty acids	*A. lebbeck *			*C. pulcherrima *			*D. mespiliformis *		
	NL	GL	PL	NL	GL	PL	NL	GL	PL
12:0	ND	ND	ND	0.30 ± 0.01	0.9 ± 0.01	0.2 ± 0.05	0.93 ± 0.10	0.72 ± 0.1	0.83 ± 0.10
14:0	ND	ND	ND	0.30 ± 0.02	0.9 ± 0.05	0.26 ± 0.01	0.90 ± 0.05	0.78 ± 0.05	0.80 ± 0.10
16:0	13.2 ± 0.50	22.3 ± 0.30	19.7 ± 0.50	17.27 ± 0.5	22.87 ± 0.50	22.4 ± 0.60	22.30 ± 0.50	22.77 ± 0.20	29.30 ± 0.50
16:1	0.7 ± 0.10	1.1 ± 0.05	ND	1.38 ± 0.05	1.14 ± 0.03	0.6 ± 0.02	0.41 ± 0.05	0.36 ± 0.05	ND
18:0	3.8 ± 0.20	6.3 ± 0.10	10.7 ± 0.10	12.40 ± 0.20	12.52 ± 0.40	9.82 ± 0.10	10.85 ± 0.20	11.71 ± 0.10	9.52 ± 0.60
18:1	19.3 ± 0.50	19.2 ± 0.30	13.5 ± 02	12.43 ± 0.10	13.87 ± 0.20	16.72 ± 0.50	24.07 ± 0.50	20.21 ± 0.20	16.70 ± 0.50
18:2	47.2 ± 0.50	36.3 ± 0.10	40.6 ± 0.10	52.81 ± 0.50	41.79 ± 0.60	48.5 ± 0.60	36.36 ± 0.30	37.83 ± 0.50	11.47 ± 0.20
18:3	0.5 ± 0.05	0.1 ± 0.20	0.3 ± 0.10	0.29 ± 0.02	0.7 ± 0.10	0.4 ± 0.02	0.54 ± 0.10	0.51 ± 0.02	26.15 ± 0.60
20:0	3.1 ± 0.10	5.0 ± 0.10	5.0 ± 0.10	1.50 ± 0.05	1.63 ± 0.02	0.8 ± 0.10	1.36 ± 0.10	1.77 ± 0.20	3.16 ± 0.40
20:1	0.5 ± 0.05	0.1 ± 0.00	0.3 ± 0.20	ND	0.53 ± 0.01	ND	ND	0.52 ± 0.01	ND
22:0	0.8 ± 0.05	0.1 ± 0.05	0.2 ± 0.20	0.82 ± 0.01	0.94 ± 0.02	0.3 ± 0.01	1.48 ± 0.20	1.54 ± 0.05	2.07 ± 0.20
22:1	6.5 ± 0.10	4.2 ± 0.10	5.1 ± 0.20	ND	ND	ND	ND	ND	ND
24:0	1.6 ± 0.20	3.6 ± 0.20	3.5 ± 0.10	0.5 ± 0.04	1.0 ± 0.02	ND	0.8 ± 0.10	1.28 ± 0.10	ND
24:1	2.8 ± 0.20	1.7 ± 0.05	1.1 ± 0.02	ND	1.21 ± 0.02		ND	ND	ND
Unsat.	77.5	62.7	60.9	66.91	59.24	66.22	61.38	59.43	54.32
Sat.	22.5	37.3	39.1	33.09	40.76	33.78	38.62	40.57	45.68

Values are mean ± standard deviation of triplicate determinations. ND = not detected, Sat. = saturated fatty acid, and Unsat. = unsaturated fatty acid.

NL = neutral lipids, GL = glycolipids, and PL = phospholipids.

**Table 4:** Unsaponifiable composition of *A. lebbeck*, *C. pulcherrima*, and *D. mespiliformis*.

*A. lebbeck *	*C. pulcherrima *	* D. mespiliformis *
Tetradecane	Beta-tocopherol	Gamma-sitosterol
Hexadecane	Vitamin E	Beta-amyrin
Phytol	Stigmasterol	Octadiene
Nonadecane	Phytol	Hexadecane
Eicosane	Octadecane	
Vitamin E		
Stigmastadiene		
Octadecane		

**Table 5:** Triacylglycerol molecular species composition (wt.%) of *B. nitida *and *G. sepium*.

ECN	Expected molecular species	*A. lebbeck *	*C. pulcherrima *	*D. mespiliformis *
C36	LnLnLn	33.27 ± 0.50	15.16 ± 0.50	ND
C38	LLnLn	33.86 ± 0.20	21.51 ± 0.80	27.13 ± 0.30
C40	LLLn/LnLnP/LnLnO	13.17 ± 0.60	42.63 ± 0.30	41.11 ± 0.50
C42	LLL/LLNO/PLLn/SLnLn	7.00 ± 0.10	7.32 ± 0.20	12.40 ± 0.20
C44	LnOO/OLL/SLLN/PLL/PLnP	7.50 ± 0.30	8.11 ± 0.30	14.40 ± 0.20
C46	POL/OOL/PLP	3.20 ± 0.50	4.27 ± 0.10	3.10 ± 0.20
C48	SLnS/OOO/POP	2.00 ± 0.20	1.00 ± 0.10	1.86 ± 0.05

Values are mean ± standard deviation of triplicate determinations. ND = not detected.

Ln = linolenic acid, L = linoleic, O = oleic acid, P = palmitic acid, and S = steric acid.
